# Momentary Peer Acceptance: An fMRI-EMA Integration Study of Peer Acceptance and Emotions

**DOI:** 10.1007/s10578-026-02007-5

**Published:** 2026-05-16

**Authors:** Mary Ryan, Timothy W. Curby, Margaret F. Bedillion, Emily B. Ansell, Tara M. Chaplin

**Affiliations:** 1 George Mason University, Fairfax, VA, USA; 2 Columbia University Irving Medical Center, New York, USA; 3 Pennsylvania State University, State College, USA

**Keywords:** Ecological Momentary Acceptance (EMA), Peer Acceptance, Functional Magnetic Resonance Imaging (fMRI), Adolescent Emotions

## Abstract

In daily life, experiences of peer acceptance play a crucial role in shaping adolescents’ emotional functioning, influencing both positive and negative emotional experiences. While prior research has linked general levels of peer acceptance to adolescent emotions using survey and laboratory-based measures, little work has examined momentary associations in naturalistic settings or investigated the neural mechanisms that may moderate these relationships. This study integrates ecological momentary assessment (EMA) and functional Magnetic Resonance Imaging (fMRI) to examine momentary associations of peer acceptance in 72 adolescents (ages 14–17) over 28 days. Results showed that momentary experiences of peer acceptance predicted higher positive emotions and lower negative emotions. Additionally, results revealed that adolescents with lower baseline temporoparietal junction (TPJ) and lower baseline anterior cingulate cortex (ACC) activation showed a stronger momentary association between peer acceptance and positive emotion. Baseline neural activation did not moderate the momentary relationship between peer acceptance and negative emotions. These findings support that momentary experiences of peer acceptance impact adolescents’ emotions in moments each day and suggests potential neural moderators for positive emotions. Findings have implications for the development of neuroscience-informed momentary peer interventions for adolescents.

## Introduction

Peer acceptance involves adolescents’ every day experiences of feeling liked, included, and valued by peers. For example, when adolescents receive friendly and responsive reactions during conversations in person and online with peers, they may experience feelings of being liked and included. Peer acceptance during adolescence promotes healthy emotional and psychological functioning, with global survey studies finding that general lower peer acceptance is associated with higher negative affect and difficulties in emotion regulation ([1-4]). Adolescents are particularly sensitive to peer acceptance because this developmental period is marked by a shift in influence from parents to peers [5].

Although several survey and lab studies have found that peer acceptance is associated with emotional benefits, these findings leave open questions about how peer acceptance influences emotional experience in moments in adolescents’ daily lives in the real world. Ecological momentary assessment (EMA) has emerged as a promising tool for capturing adolescents’ real-time emotional reactions to social experiences, revealing dynamic associations between momentary experiences of peer acceptance and momentary positive and negative emotions [6-8]. The current study used EMA to examine momentary within-person associations between experiencing peer acceptance and positive and negative emotions in adolescents. Additionally, the present study explored adolescents’ neural responses to peer acceptance (measured in fMRI) at baseline as a moderator of momentary associations between peer acceptance and emotions. These findings may increase our understanding of adolescent emotional functioning in the presence of peer interactions, and inform future research on adolescent interventions, including momentary interventions.

### Peer Acceptance and Adolescent Emotions: Behavioral Studies

Research on adolescent emotional experiences, and what may impact emotions, is important, as the presence of higher positive affect and lower negative affect have been associated with lower depressive, anxiety, and other psychological symptoms in youth [9]. Lack of peer acceptance appears to be maladaptive for adolescents, as lower general levels of peer acceptance (e.g., peer acceptance measured using peer ratings) has been associated with greater general levels of negative emotions (measured at the same time as peer acceptance) in adolescent self-report and parent report global questionnaire studies [1, 2]. On the other hand, greater peer acceptance appears to be adaptive for adolescents, as greater peer acceptance is associated with greater positive emotions and lower depressive symptoms and social anxiety symptoms in adolescents in survey studies that measure general peer acceptance and past month or two symptoms and lab studies that induce peer acceptance experimentally ([3, 4, 10, 11]). Peer acceptance may have a particularly high impact on emotionality during early to middle adolescence (relative to childhood), given greater emphasis on peer relationships and heightened activation of social networks in the brain [12]. While studies of global questionnaire measures and lab based studies of responses to controlled peer acceptance stimuli seem to indicate associations between peer acceptance and emotions in youth, further research is needed to understand how these associations unfold in moment-to-moment time frames and in real-world contexts.

### Peer Acceptance and Adolescent Emotions: EMA Studies

Ecological Momentary Assessment (EMA), or intensive repeated sampling in-vivo, is a research method that captures individuals’ real-time emotions, behaviors, and experiences in their everyday settings. This approach allows researchers to examine how experiences fluctuate within individuals over time, offering insights that traditional long-term retrospective methods may miss.

There have been a few initial EMA studies of peer interactions and emotions in adolescents. Most of these studies have focused on experiences of peer rejection, typically finding that greater momentary peer rejection is associated with greater negative emotion and lower positive emotion (e.g., [13-15]). Only three EMA studies examined aspects of peer acceptance and emotions in adolescents.

One EMA study that measured peer interactions and emotions 3–5 times per day found that momentary increases in the number of interactions with peers that were perceived as popular predicted greater reported momentary positive affect in adolescents age 11–13 [8]. Another EMA study of adolescent girls age 11–13 that measured peer interactions and emotions 3–4 times per day found that momentary experiencing a greater number of positive peer interactions was related to higher momentary positive emotion and that this association was stronger for in-person peer acceptance versus social media peer acceptance [7]. The Hamilton et al., [7] and Rusby et al., (2012) findings suggest that greater momentary peer acceptance predicts greater concurrent positive emotions. Findings from another study suggest that lower momentary peer acceptance may contribute to negative emotions. Ha et al., [6] found that lower levels of feelings of being accepted by peers (and higher feelings of peer rejection) in the moment were associated with greater negative emotions and lower positive emotions in 13 to 16 year olds. In sum, a small body of literature has begun to examine peer acceptance in EMA, and has found that peer acceptance may support adolescent positive emotions and reduce adolescent negative emotions in the moment.

### Neural Responses Moderating EMA Associations

In addition to understanding momentary effects of peer acceptance on adolescents’ emotions, it may also be important to identify factors that may make adolescents more or less susceptible to the daily impact of peer acceptance. This can help us to understand for whom momentary peer acceptance matters most. One potential moderating factor may be adolescent’s neural sensitivity to peer acceptance. A few studies have begun to examine the moderating role of neural sensitivity to peer acceptance and have found a role in particular for neural responses in anterior cingulate cortex (ACC) and the temporal parietal juncture (TPJ).

Guyer and colleagues [16] found that adolescents (age 9–17) experiencing peer acceptance in a chatroom fMRI task were more likely to report greater positive emotions directly after the fMRI task when they also had higher activation in the anterior cingulate cortex (ACC) to peer acceptance. Thus, in this study, ACC activation was a neural moderator that strengthened the relationship between lab-simulated peer acceptance and self-report positive emotions. The ACC helps detect and evaluate emotionally and socially relevant stimuli and can be especially important for helping adolescents determine what matters emotionally and socially in their environment [17]. Building on this, adolescents with heightened ACC activation may be more attuned to the emotional salience of peer feedback, which could amplify their emotional responses to moments of acceptance. In line with this idea, Silk and colleagues [18] examined how ACC activation relates to adolescents’ experiences of peer closeness in daily life using ecological momentary assessment (EMA). In a 10-day EMA study with 38 adolescents (ages 11 to 16), they found that greater ACC activation in response to peer acceptance stimuli during fMRI was associated with higher average feelings of closeness to peers in daily life [18]. Given that the ACC helps detect and evaluate emotionally and socially relevant stimuli, this finding may indicate that youth who show salience responses to (and find peer acceptance salient/rewarding) may have more benefits of experiencing peer acceptance in daily life. A next step in this line of research would be to test ACC responses to peer acceptance as moderators of within-person momentary associations between peer acceptance and emotions in daily life. The present study will examine this.

In addition to ACC responses, one study found TPJ responses to peer acceptance in fMRI to be associated with emotional response to peer acceptance in EMA. Flores et al., [19] examined the moderating effects of TPJ responses in fMRI on the relationship between momentary emotional closeness with friends and positive emotions in a sample of 34 adolescents (age 14–18). They found that greater in-the-moment report of emotional closeness was more strongly associated with momentary higher positive emotion for adolescents that had lower activation in the right TPJ in response to peer acceptance stimuli [19]. The TPJ is involved in social cognition and perspective-taking [20]. Findings from Flores et al., [19] may suggest that adolescents with lower TPJ activation are less focused on cognitively evaluating peer acceptance and are allowing affective experience of it. These youth may be more emotionally available in the moments of peer acceptance, allowing them to more fully experience the positive effects of emotional closeness with others rather than over-evaluating the interactions. Thus, overall, initial studies have begun to integrate fMRI and EMA to examine links between neural sensitivity, in-the-moment peer acceptance social feedback, and real-world emotional experiences, which bridge internally-consistent controlled fMRI findings with more ecologically-valid daily processes and to help understand for whom momentary social acceptance is salient for emotional functioning.

### Present Study

The present study adds to the existing literature in two ways. First, using EMA, the study contributes to a small body of work that has begun to test if the between-person associations between general levels of peer acceptance or lab-induced peer acceptance and positive/negative emotion found in survey and lab studies exist in within-person associations in daily moments in real world settings. Second, the present study is one of the first to examine neural moderators of momentary effects of peer acceptance, which can advance our understanding of which youth benefit the most from momentary peer acceptance. The present study tests two sets of hypotheses:

**Hypothesis 1a:** *We predict that greater within-person momentary peer acceptance will predict momentary increases in positive emotion*.
**Hypothesis 1b** *(TPJ): We predict that lower neural TPJ activation to peer acceptance in fMRI will strengthen the relationship between greater momentary peer acceptance and momentary positive emotion*.
**Hypothesis 1c** *(ACC): We predict that greater neural activation in the anterior cingulate cortex (ACC) will strengthen the relationship between greater momentary peer acceptance and momentary positive emotion*.
**Hypothesis 2a:** *We predict that greater within-person momentary peer acceptance will predict momentary decreases in negative emotion*.
**Hypothesis 2b** *(TPJ): We predict that lower baseline neural activation in the TPJ will strengthen the relationship between greater momentary peer acceptance and lower momentary negative emotion*.
**Hypothesis 2c** *(ACC): We predict that greater neural activation in the ACC will strengthen the relationship between greater momentary peer acceptance and lower momentary negative emotion.*

## Methods

### Participants

This study draws on a sub-sample of 96 adolescents selected from a larger longitudinal study on adolescent risk behaviors, involving 248 youth (i.e., PAIT; citation omitted for blind review). Participants in this sub-sample were recruited based on elevated risk profiles, including a history of substance use or elevated maladaptive parenting behavior. The fMRI was conducted at baseline, when youth were aged 11–14. The ecological momentary assessment (EMA) was conducted at the sub-sample participants’ 2- or 3-year follow-up visit (i.e., 2–3 years after the baseline session), when participants were between 14 and 17 years old (M = 15.63, SD = 0.88). The EMA spanned 28 days. Of the 96 adolescents recruited for the EMA, 84 completed the full EMA protocol. Of these 84, 72 adolescents had usuable fMRI data for the peer acceptance fMRI task at baseline (8 had excessive motion- see below, 4 did not complete the fMRI peer acceptance task) (for demographic information, see Table 1).

### EMA Training

Participants completed a 45-minute orientation during which a research assistant guided them through the EMA procedures. This included downloading the survey app, creating a username and password, and adjusting phone settings to enable survey notifications. Participants then completed a test survey on their phone, during which each EMA item was reviewed for clarity and rating instructions were provided (e.g., using the full 0–100 scale for affect ratings, responding promptly, and answering honestly). To promote compliance, participants received $1 per completed survey and a $25 weekly bonus for completing at least 85% of surveys. Daily text reminders informed participants of their current compliance and earnings. Participants were also reminded to keep their phones charged to ensure survey delivery. These procedures were based on prior EMA protocols with demonstrated success in maximizing compliance (e.g., [21]).

#### EMA Compliance

Participants included in the study completed 40% or more of the daily assessments. Prior studies of adolescents used inclusion cutoffs that ranged from 20% [6] to 50% [18]. Overall compliance was 74%. Based on a recent meta-analysis on compliance for child and adolescent EMA, studies with greater than 70% compliance are considered to be “good” samples, as studies prompting 5 or more times per day over longer durations (e.g., > 21 days) tended to see compliance between 54 and 96%, with an average of 74.4% [22].

### EMA Procedures and Measures

For the EMA portion, adolescents completed surveys at 5 timepoints throughout the day for 28 days. The first survey appeared for participants at 7am, and participants were instructed to complete it when they woke up. Surveys 2, 3, and 4 were sent out at a randomized time within the intervals of 3-5pm, 5-7pm, and 7-9pm. These surveys were programmed so that they were spaced out by at least 90 min. The fifth and final survey was sent out at 9:30pm and participants were instructed to complete it at their bedtime. Participants completed surveys through MetricWire, a secure smartphone application [23]. Each survey included questions on emotional arousal (both positive and negative), and questions on positive peer interactions adapted from the Perceived Acceptance Scale (PAS; [24]) in addition to other questions as part of a larger study.

#### Emotions

Adolescents’ emotions were measured in-the-moment using the language “*right now*” and were rated on a 0–100 Visual Analogue Scale (VAS), where “0” indicated the lowest amount of the emotion and “100” indicates the highest amount of the emotion. Positive emotion questions were “*Right now*, *how happy do you feel?*”, and “*Right now*, *how excited do you feel?*”. Ratings from these questions were averaged together to create the positive emotion variable. Negative emotion questions were “*Right now*, *how sad do you feel?*”, “*Right now*, *how upset do you feel?*”, “*Right now*, *how mad do you feel?*”, “*Right now*, *how annoyed do you feel?*”, and “*Right now*, *how stressed do you feel?*”. These questions were also averaged together to create the negative emotion variable. These items were from the PANAS child [25], which has been used in EMA studies of adolescents [26].

#### Experience of peer acceptance

Peer acceptance in the present study was assessed with one dichotomous question: “*Since last survey, I felt accepted by a peer my age*” where the response options were “*yes*” or “*no*”. This item is similar to ones used in prior EMA studies of peer acceptance [7]. Participants were instructed to select “*yes*” when they had experienced a positive peer interaction. When adolescents responded yes to this question, they reported on how long ago the event occurred, how long it lasted, which peer it was with, and how did they interact. In the present study, our measure of peer acceptance is yes (1) or no (0) on whether they experienced peer acceptance since last survey. Peer-acceptance items were intended to reflect experiences since the prior survey, though timing may have been misestimated or carried forward. We conducted a sensitivity check comparing adolescents’ “*how long ago did this happen?*” responses to the interval since the last survey; 44 surveys (0.51%) fell outside the intended window. Excluding these surveys did not meaningfully change the primary results.

### fMRI Procedures

#### Peer Acceptance fMRI Task

At a baseline session two weeks before the MRI, adolescents rated 40 peer photographs based on the following instructions: “*please rate how much you like or dislike the teen in the photo*” (1 = not at all, 9 = very much). The peer photographs were actually images from a standardized database and community actors. Participants were then photographed themselves and told their image would be rated by other teens in the study.

At the MRI session, participants were reminded of the task and told they’d see photos of peers they had rated, along with symbols indicating whether those peers had “liked” them (thumbs up) or not yet rated them (“neutral” condition, yellow circle). In reality, other teens in the study did not rate them. During the ~ 6.7-minute task, participants viewed 40 photos of peers (selected for the peers that they had rated highly or moderately)—of the photos, 20 were paired with “likes” (thumbs up symbol), and 20 were marked as “not yet rated” (a yellow circle). Photos were presented in a randomized, event-related design with 3-second displays and jittered interstimulus intervals. The task was adapted from validated adolescent peer social evaluation paradigms [12, 16]. Participants were debriefed afterward and informed that the peer images were actors and their own photo had not been shared.

#### fMRI Analysis

FSL’s fMRI Expert Analysis Tool (FEAT) was used to conduct first-level analyses using a temporal and spatially regularized autocorrelation model. Contrasts of interest were generated by comparing the “Like” versus “Neutral” trials in the peer acceptance task, based on the Coefficient of Parameter Estimate (COPE) values for each explanatory variable. Adolescents were not included in the present study if they exceeded motion thresholds in the peer acceptance task: more than 20% of TRs with framewise displacement (FD) > 0.5 mm or more than 5% of TRs with FD > 3 mm (*n* = 8). These standards are consistent with acceptable parameters in adolescent fMRI studies [27].

Regions of interest (ROIs) were the right (R) and left (L) anterior cingulate cortex (ACC) and (R) and left (L) temporoparietal junction (TPJ) (see Table 2). In selecting ROIs we included left and right separately, given that function may differ by hemisphere for these regions [28]. ROI masks were created by first identifying peak MNI coordinates for each region using the Automated Anatomical Labeling (AAL) atlas in MNI standard space [29]. Around each peak coordinate, a 10 mm radius spherical mask was generated. To ensure that the extracted signal reflected gray matter activation, each spherical mask was then multiplied by the MNI152 gray matter tissue probability mask, effectively excluding white matter and cerebrospinal fluid.

Mean activation values (COPE values) were extracted from each resulting ROI mask, representing average BOLD signal within the defined region. These values were then used in subsequent group-level analyses.

### Statistical Analysis

Analyses were conducted using R Statistical Software (R Core Team, 2022). Linear mixed-effects models were estimated using the *lmer* function from the *lme4* package [30]. These models account for the nested structure of the data, where momentary assessments (Level 1) were nested within individuals (Level 2). All models were estimated using restricted maximum likelihood (REML). All models included fixed effects to control for age, sex, study day (to account for time trends), and whether the report occurred on a weekend (vs. weekday) and with continuous predictor variables centered. To separate within-person and between-person effects, peer acceptance effects were decomposed into two components: a within-person (person-mean centered) variable at level 1 and a between person (grand-mean centered) variable at level 2. This allows examination of momentary within-person effects of peer acceptance on emotion, controlling for averaged between-person associations between peer acceptance and emotion.

Models included a random intercept to allow for variability across individuals in mean positive and negative emotion levels. Models also included a random effect (random slope) for within-person peer acceptance, which allows for variability across individuals in the within-person peer acceptance to emotion associations over time. Finally, models included a random effect of day in study to allow for variability in the effect of study day on emotion.

To examine within-person associations between peer acceptance and positive and negative emotional experiences, we conducted two multi-level models. The first model tested whether momentary peer acceptance was associated with positive emotion (Hyp 1a) and the second tested whether momentary peer acceptance was associated with negative emotion (Hyp 2a). Results from these models would represent recent experiences of peer acceptance being associated with current emotion. Given that peer acceptance at each assessment was reported as occurring “since the last survey” and emotions were reported “right now,” models were concurrent rather than lagged. Results from the model would represent recent experiences of peer acceptance being associated with current emotion.

To examine moderation by neural activation, we conducted eight models, with separate models for each type of emotion (positive, negative) and for each neural ROI moderator (R TPJ, L TPJ, R ACC, L ACC). To account for multiple comparisons across the four positive emotion and four negative emotion models testing neural moderators, we applied a false discovery rate (FDR) correction using the Benjamini-Hochberg procedure [31]. Hypotheses 1b and 2b tested whether activation in the R and L TPJ at baseline moderated the relationship between momentary peer acceptance and positive emotions (Hyp 1b) and negative emotions (Hyp 2b). Hypotheses 1c and 2c tested analogous moderation effects for R and L ACC. Each model included within-person momentary peer acceptance at Level 1, neural activation at Level 2, and cross-level interaction terms between peer acceptance and neural activation.

#### Data Cleaning

Prior to conducting multilevel modeling, data were examined for outliers and distributional assumptions. For continuous variables, values exceeding ± 3 standard deviations from the sample mean were winsorized to the nearest non-outlier value to reduce the influence of extreme cases while maintaining the overall distribution. Only the positive and negative emotion variables were winsorized.

Variables were then assessed for normality, linearity, multicollinearity, and homoscedasticity. Visual inspection of histograms and Q-Q plots indicated that the distributions of positive and negative emotion scores, as well as the baseline neural activation values (COPEs from ACC and TPJ ROIs), were approximately normal. Skewness and kurtosis statistics were within acceptable bounds (± 1.0) for most variables, and none exceeded ± 2.0. One neural region (L TPJ) showed slightly elevated kurtosis, but remained within acceptable thresholds for parametric analyses [32].

## Results

### Hypothesis 1a: Peer Acceptance Main Effect on Positive Emotions

Results of the model for within-person momentary peer acceptance predicting positive emotions revealed a significant main effect of peer acceptance, β = 0.07, SE = 0.65, t(6946) = 8.12, *p* <.001 (see Table 3), indicating that experiencing recent peer acceptance (reported since the last survey) was associated with higher positive emotion (reported currently). This means that adolescents that had experienced instances of peer acceptance recently reported greater positive emotions.

In addition, we found associations for two of the other variables. Specifically, we found that between-subjects peer acceptance was associated with positive emotions, such that individuals with higher average levels of peer acceptance reported increases in momentary positive emotions. Additionally, we observed an effect of day of study on positive emotion, with positive emotion increasing over the course of the 28 day EMA. Weekday versus weekend status, age, and sex were not significantly associated with positive emotions.

### Hypothesis 1b: Peer Acceptance by TPJ Activation on Positive Emotions

We tested whether baseline neural activation in the L and R TPJ moderated the momentary within-person association between peer acceptance and positive emotion in two separate models. In the model including L TPJ, the interaction between peer acceptance and L TPJ activation was significant (β = −0.05, *t*(65.61) = −3.23, *p* =.002), indicating that L TPJ activation moderated the effect of within-person peer acceptance on positive emotion (see Table 4). In the model including R TPJ, the interaction term was also significant (β = −0.04, *t*(69.64) = −2.61, *p* =.011), suggesting a similar moderating effect for right TPJ activation (see Table 5).

To interpret these interactions, we plotted the simple slopes of the association between peer acceptance and positive emotion at high (+ 1 SD) and low (−1 SD) levels of TPJ activation (see Fig. 1). In both models, adolescents with *lower* TPJ activation—left or right—demonstrated a stronger association between higher within-person peer acceptance and higher positive emotion. These results suggest that adolescents with lower neural sensitivity in TPJ regions may experience greater emotional benefits from peer acceptance in daily life.

### Hypothesis 1c: Peer Acceptance by ACC Activation on Positive Emotions

We tested whether baseline neural activation in the L and R ACC moderated the momentary within-person association between momentary peer acceptance and positive emotion in two separate models. In the model including L ACC, the interaction between peer acceptance and L ACC activation was significant (β = −0.04, *t*(75.65) = −2.61, *p* =.001), indicating that L ACC activation moderated the effect of within-person peer acceptance on positive emotion (see Table 6). In the model including R ACC, the interaction term was also significant (*β* = −0.03, *t*(73.17) = −2.06, *p* =.043) suggesting a similar moderating effect for R ACC activation (see Table 7).

To interpret these interactions, we plotted the simple slopes of the association between peer acceptance and positive emotion at high (+ 1 SD) and low (−1 SD) levels of TPJ activation (see Fig. 2). In both models, adolescents with lower ACC activation—left or right—demonstrated a stronger association between higher within-person peer acceptance and higher positive emotion. These results suggest that adolescents with lower neural sensitivity in ACC regions may experience greater emotional benefits from peer acceptance in daily life.

### Hypothesis 2a: Peer Acceptance and Negative Emotions

Results of the model for momentary within-person peer acceptance predicting negative emotions in the EMA revealed a significant main effect of peer acceptance (β = −0.56, *SE* = 0.42, *t*(6801) = −7.28, *p* <.001; see Table 8) indicating that peer acceptance since the last survey was associated with lower current negative emotion. This means that adolescents who had experienced instances of peer acceptance recently reported lower negative emotions.

In addition, we found some significant associations for covariate variables (see Table 8). Weekday versus weekend status predicted negative emotions, with lower negative emotions on weekends. Sex was also a significant predictor, with girls reporting higher levels of negative emotion than boys. Age and study day were not significantly associated with negative emotion. Finally, between-subjects mean level of peer acceptance did not predict negative emotions.

### Hypothesis 2b: Peer Acceptance by TPJ Activation on Negative Emotions

Results of the models testing the interaction between within-person peer acceptance at level 1 and between-person TPJ activation at level 2 predicting negative emotion revealed no significant peer acceptance X TPJ interaction for the L TPJ or R TPJ. This indicated that L TPJ and R TPJ activation did not moderate the within-person relationship between peer acceptance and negative emotions in the EMA. Additionally, there was also no main effect of L or R TPJ activation on negative emotion.

### Hypothesis 2c: Peer Acceptance by ACC Activation on Negative Emotions

Results of the models testing the interaction between within-person peer acceptance at level 1 and between-person ACC activation at level 2 predicting negative emotion revealed no significant peer acceptance X ACC interaction for the L ACC or R ACC. This indicated that L ACC and R ACC activation did not moderate the within-person relationship between peer acceptance and negative emotions in the EMA. Additionally, there was also no main effect of L or R ACC activation on negative emotion.

## Discussion

The goal of this study was to understand the momentary associations between peer acceptance and adolescent emotional experiences in daily life using EMA, and to test whether neural responses to peer acceptance in fMRI moderated these associations. Our results suggest that experiencing peer acceptance in real-time was associated with increased positive emotions and decreased negative emotions in adolescents, supporting the hypotheses that peer acceptance enhances positive emotion and reduces negative emotion in moments in daily life. This process is important, as these repeated interactions over days may increase socio-emotional well-being over time. Additionally, neural activation in the ACC and TPJ moderated the within-person effects of momentary peer acceptance on positive emotion (though not negative emotion), suggesting that individual differences in neural sensitivity to peer acceptance may shape the extent to which peer acceptance in daily life impacts adolescents’ positive emotional states. In sum, our results suggest that momentary experiences of peer acceptance in daily life are important for adolescents’ positive and negative emotions and, for positive emotions, that may be more true for adolescents that show lower neural reactivity to peer acceptance.

### Momentary Peer Acceptance Effects on Positive Emotions

We hypothesized that momentary experiences of peer acceptance would predict greater positive emotions, and, consistent with this, we found that adolescents reported significantly greater positive emotions in moments when they had recently experienced acceptance by their peers. These results emerged at the within-person level, indicating that fluctuations in peer acceptance on a moment-to-moment basis predicted corresponding changes in positive emotional states. Our results are consistent with previous non-EMA studies finding that between person general peer acceptance levels are associated with global high positive emotions [3, 4]. Our results support these findings and extend them into within-person impacts of peer acceptance on positive emotions in moments in real world settings.

Importantly, the peer acceptance item within our study reflects adolescents’ perceived acceptance since the prior prompt, rather than an objective record of discrete number of peer events. As a result, the observed associations may reflect not only the occurrence of a positive interaction, but also adolescents’ attention to, interpretation of, and appraisal of peer experiences as accepting—processes that may be especially salient when interactions are ambiguous. Future work examining peer acceptance in EMA should consider both perceived acceptance and discrete number of experiences teens report by incorporating event-contingent sampling and brief event-level follow-ups (e.g., describing distinct acceptance experiences, and rating perceived acceptance intensity/meaning), alongside prompt-based ratings.

Our finding of momentary peer acceptance predicting positive emotion in EMA is also consistent with and builds upon the few prior EMA studies of peer acceptance (e.g., [7, 8]) that found adolescents report greater positive affect during moments of perceived peer acceptance. Our study also extends this literature, which has focused on early adolescence (ages 11–13), into middle adolescence, demonstrating that the momentary emotional benefits of peer acceptance persist into middle adolescence (ages 14–17), a developmental period marked by heightened salience of peer relationships and increased autonomy. Taken together, these EMA findings have several implications. First, momentary experiences of peer acceptance may function as immediate emotional boosts occurring in daily life and these may accumulate over time, which may support adolescents’ increased emotional well-being. Also, as we know that momentary higher positive affect is associated with lower depressive and anxiety symptoms in youth [9], peer acceptance and positive emotion may also buffer youth from experiencing depression or other psychopathology symptoms. Our results thus may have meaningful implications for interventions: strategies that increase opportunities for daily peer acceptance—such as social skills training, peer mentorship, or group-based programs—may help enhance positive emotions in youth. Finally, our findings establish that peer acceptance and positive emotions are associated in EMA, which points to the potential utility for momentary or just-in-time adaptive interventions that target social experiences (or emotional reactions to those experiences) in real-time to promote emotional resilience during adolescence.

### Peer Acceptance by Neural Activation on Positive Emotions

#### TPJ Findings

We also found that youth with lower baseline TPJ activation showed a stronger association between peer acceptance and positive emotion in the moment. This pattern was consistent with our hypothesis and replicated findings from Flores et al., [19], who found that emotional closeness with friends was more strongly associated with positive affect in EMA for adolescents with lower TPJ activation to peer acceptance in fMRI. Given that the TPJ is primarily involved in social cognitive processes such as perspective-taking and interpreting others’ intentions [20], it is plausible that adolescents with higher TPJ activation may be more engaged in cognitive evaluation of social interactions, which could blunt their positive emotional responsiveness in the moment. In contrast, youth with lower TPJ activation may be more attuned to allowing the affective aspects of peer interactions, enabling them to have for more emotionally rich and rewarding experiences. It is notable that both Flores et al., [19] and our study found a role for the TPJ and this may indicate that the TPJ is an important region for moderating the effect peer acceptance on positive emotions. These findings—across both studies—suggest that adolescents with lower TPJ activation may benefit from peer acceptance interventions in a unique way. For example, these adolescents might respond especially well to interventions that emphasize positive peer feedback in emotionally immersive or experiential formats, rather than those requiring high levels of cognitive appraisal or social reasoning. Strategies such as social reward priming, emotion-focused group interactions, or real-time affirmations in peer settings (e.g., positive text messages, group chats, or peer compliments) may be particularly effective in enhancing mood and well-being. Future research should test whether adolescents with lower TPJ activation show greater gains from such emotions-focused peer interventions compared to those involving more cognitively mediated approaches (e.g., perspective-taking or structured peer evaluations).

#### ACC Findings

The present study also found that baseline ACC activation moderated the association between peer acceptance and positive emotion, though this was in the opposite direction than expected. While we anticipated that greater ACC activation would amplify the emotional benefits of peer acceptance—consistent with Guyer et al., [16], who found increased positive affect following peer acceptance in a between-persons lab study among adolescents with greater ACC activation—we found that lower ACC activation was associated increased positive affect following peer acceptance in EMA. This suggests that adolescents with reduced ACC engagement during social reward may be more emotionally receptive to peer acceptance at a within-person level in moments in daily life. One interpretation is that lower ACC activation reflects youth that typically have a blunted salience response to peer stimuli and suggests that, for these blunted youth, experiencing peer acceptance in moments in daily life gives them a momentary boost in positive emotions. In this context, lower ACC activation may represent an adaptive neural response, promoting greater emotional benefit from peer interactions in everyday life. Of course, our study only focused on two hypothesized regions in the brain and future studies that use whole brain approaches may be helpful to understand if this blunted neural reactivity extends to other regions.

### Peer Acceptance and Negative Emotions

We predicted that experiencing peer acceptance in daily life would be associated with lower negative emotions. Consistent with previous between-person non-EMA research finding that global peer acceptance is correlated with global lower negative emotion (e.g., Woodhouse et al., 2011), adolescents in our EMA study reported significantly lower negative emotions in moments when they had recently experienced peer acceptance. Our finding is consistent with the one prior EMA study on peer acceptance and negative emotion [6] which found that lower levels of peer acceptance (and higher peer rejection) in the moment were associated with greater negative emotions in middle adolescence. Taken together, these two momentary studies extend the prior between-person survey studies to find that peer acceptance is important at a within-person level in moments in everyday life. This indicates that effects of peer acceptance unfold over moments within adolescents and that momentary fluctuations in peer acceptance are important in decreasing negative emotional states.

Our results linking peer acceptance to lower negative emotions emphasize the importance of fostering peer acceptance during adolescence. Since lower negative emotion is associated with lower incidence of depression and anxiety in adolescence [33], it is important to understand what leads adolescents to experience lower negative emotions. Thus, our findings suggest that peer acceptance in daily life lead to lower negative emotions in the immediate moment and thus may over time protect youth from experiencing internalizing symptoms. Interventions that create frequent, meaningful peer interactions—such as peer-led programs or structured group activities—may be especially effective in reducing adolescents’ momentary distress and supporting overall emotional well-being. Interventions could also utilize EMA by prompting adolescents to reflect on or seek out positive peer interactions in real time. This strategy may offer a promising way to support emotional well-being by leveraging everyday social experiences.

### Peer Acceptance by Neural Activation on Negative Emotions

Unlike the findings for positive emotion, neural activation did not significantly moderate the association between peer acceptance and negative emotion. Specifically, neither TPJ nor ACC activation influenced the strength of the relationship between momentary peer acceptance and negative mood. These results suggest that while adolescents generally experience emotional relief of negative emotions following peer acceptance, this effect may be driven by more basic or universally reinforcing mechanisms—such as felt belonging or social safety—rather than by individual differences in neural reactivity, at least in these salience ROIs. In contrast, positive emotional responses to peer acceptance may require more individualized processing, such as reward sensitivity or social salience detection, which vary by individual. Alternately, it could be due to lower power for the fMRI task to moderate associations given that the MRI data was collected 2–3 years before the EMA data (and MRI responses likely change developmentally over that period).

Future studies should explore whether neural activation plays a more central moderating role in the context of peer rejection or exclusion associations with negative emotions, where individual differences in vigilance, emotion regulation, and cognitive appraisal may have a greater impact on negative affective outcomes (e.g., [18]). Broadening the scope to examine both peer acceptance and peer rejection experiences in daily life EMA studies may clarify whether brain regions like the ACC and TPJ are context-sensitive—or whether they play general roles in social affective processing across both positive and negative interpersonal events.

### Examining Contextual and Developmental Considerations

Beyond examining neural moderation, our results also revealed significant contextual effects on adolescent’s momentary emotions in daily life. Adolescents reported greater negative emotions on weekdays compared to weekends, suggesting that emotional experiences may be shaped by broader environmental factors and social opportunities. This finding is consistent with prior research which found improvements in mood on weekends versus weekdays due to increased sleep ([34, 35]). Our findings highlight the importance of examining differences in adolescent emotions on weekends versus weekdays.

Additionally, gender differences emerged, with girls reporting higher negative emotions overall. Prior research has also found that adolescent girls express greater negative emotions than adolescent boys [36]. This pattern may reflect, in part, the higher prevalence of depression and anxiety among adolescent girls ([37]), as well as gender-linked developmental processes (e.g., pubertal timing, gender role socialization, social-evaluative concerns, rumination on negative experiences, and interpersonal stress) that can amplify negative emotional experiences during this period particularly for girls ([38-40]).

Overall, these findings underscore the importance of considering contextual and demographic factors when examining adolescent emotional experiences, and may help inform interventions. Additionally, momentary interventions could track behaviors such as ruminative thinking seen more often in girls, in order to develop more effective coping strategies to process negative experiences and reduce negative emotional experiences.

### Limitations and Future Directions

The present study advanced the existing literature by examining the relationship between peer acceptance and adolescent emotion in moments in real world contexts and by innovatively testing neural sensitivity as a moderator of momentary effects of peer acceptance. Despite the strengths of this study, several limitations should be acknowledged. First, while EMA provides a valuable real-world assessment of momentary peer acceptance and emotions, our EMA was limited to self-reported experiences only, which may be subject to biases such as recall errors (though the intensive sampling of EMA reduces these biases). Future studies could incorporate passive sensing methods or peer-reported measures of social acceptance to enhance validity. Another limitation is the time gap between the baseline fMRI and EMA follow-up. Neural activation was measured 2–3 years prior to the EMA, and because ACC and TPJ functioning may shift across adolescence ([41, 42]), neural responding may have changed between assessments. This mismatch may have reduced power to detect moderation effects and limits conclusions about neural activation and momentary emotions operating within the same time window. Future work should assess fMRI and EMA closer in time or use repeated neuroimaging across adolescence to clarify how developmental changes in neural sensitivity relate to adolescents’ momentary emotional experiences. Additionally the study sample characteristics should be acknowledged. First, our sample was predominantly White, which may limit the generalizability of findings to adolescents from diverse cultural backgrounds. Cultural values can meaningfully shape adolescent emotional and neural responses to social experiences, including peer acceptance and future research with larger more diverse samples can examine this. Relatedly, our recruitment strategy focused on including families with an elevated-risk profile. This strategy allowed for an important higher risk group in which to explore peer acceptance and emotion. However, findings may not generalize to lower-risk community adolescents. This is especially important to consider within our TPJ findings as a prior study found that TPJ functional connectivity was altered in children exposed to higher family stress ([43]).

Finally, while the present study captured the impacts of peer acceptance on adolescent emotion, which is relatively understudied compared to peer rejection, our study could have been improved by measuring fMRI (and EMA) responses to both peer acceptance and peer rejection. Interestingly, one study examined neural responses to peer rejection as moderators and found a unique role for the TPJ when responding to rejection versus acceptance [44].

## Conclusion

Overall, this study provides novel insights into the dynamic momentary relationship between peer acceptance and adolescent emotions through use of EMA. It also uniquely integrated real-world EMA data with fMRI neural correlates of social processing. Our findings suggest that momentary experiences of peer acceptance play an important role in shaping adolescents’ momentary positive and negative emotions *in daily life* and that neural sensitivity in ACC and TPJ moderate this role for positive emotions. Our results highlight the relevance of considering both real-time social experiences and individual differences in neural processing when studying adolescent social functioning. Future research should continue to explore these interactions to inform interventions aimed at promoting positive peer experiences and emotional resilience in youth. By bridging brain and behavior, our study advances our understanding of how adolescents’ social environments and neural responses jointly shape social-emotional functioning for youth, and point to the potential of neuro-informed, real-time research to support youth during this pivotal developmental window.

## Figures and Tables

**Fig. 1:**
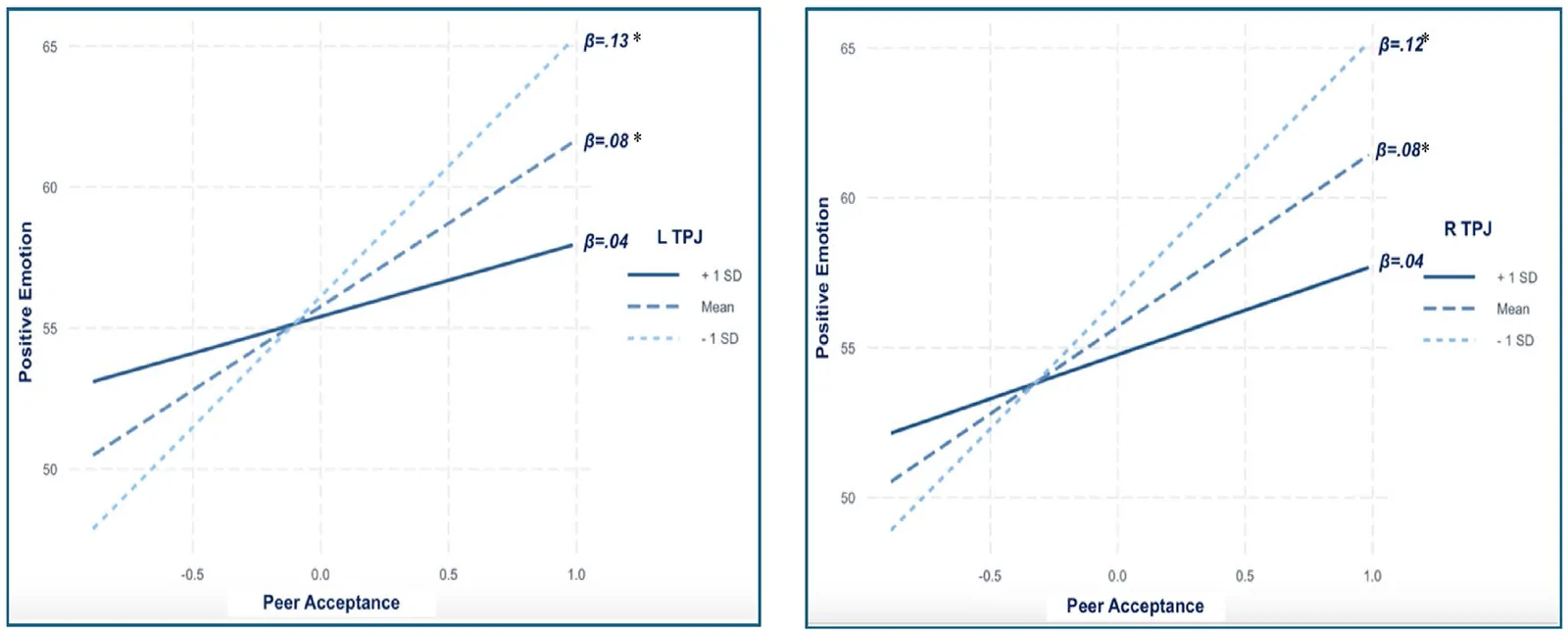
Simple slopes plotted for L TPJ and RTPJ

**Fig. 2:**
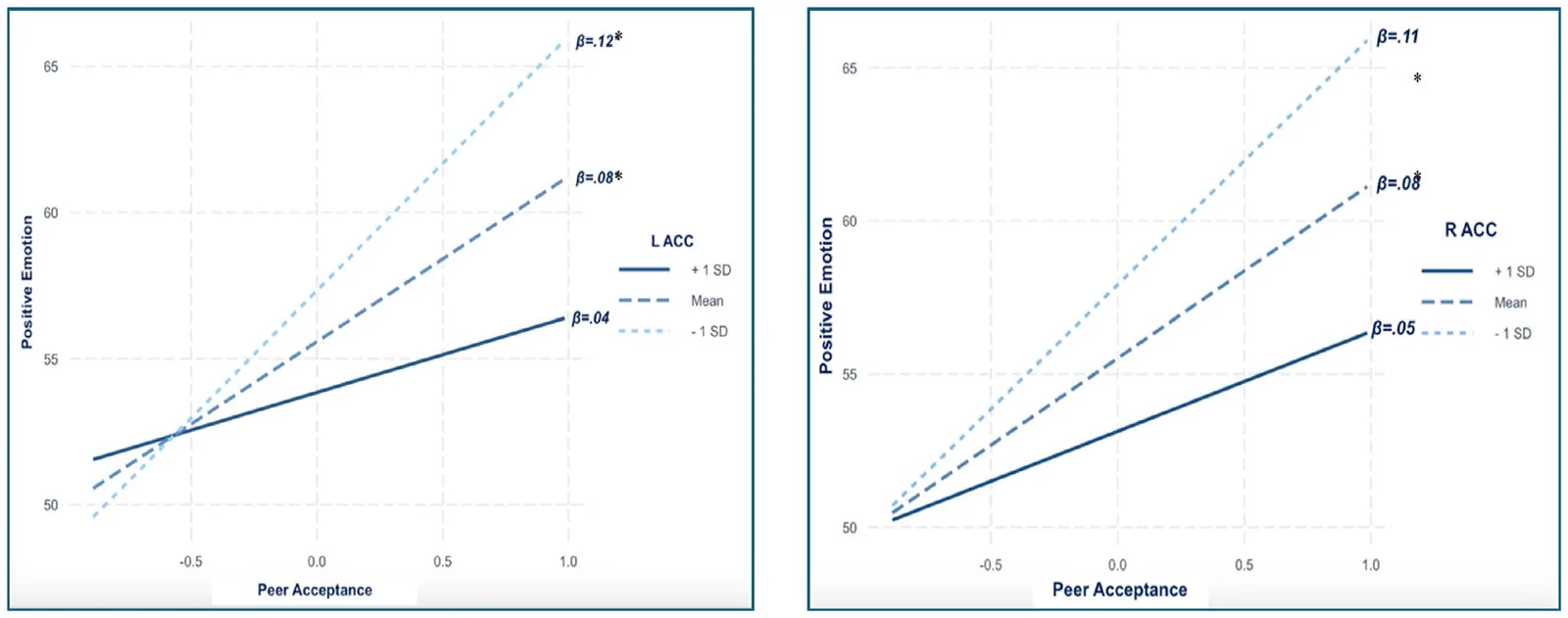
Simple slopes plotted for L ACC and R ACC

**Table 1:** Demographic Information and Descriptive Statistics for Study Variables

Age (years)	Mean (or %)	s.d	Range
	15.63	0.88	14–17
**Sex (Male/Female)**	53%/47%		
**Race/Ethnicity**			
**White**	58%		
**Hispanic**	14%		
**Mixed Race**	9.6%		
**Black**	8.4%		
**Asian**	6.1%		
**Other**	3.9%		
**Positive Emotions**	56.29	24.62	0–100
**Negative Emotions**	20.31	18.39	0–100

**Table 2:** Coordinates for ACC and TPJ

Hemisphere	Region	MNI Coordinates (x, y, z)
Left	ACC	−6, 38, 20
Right	ACC	6, 38, 20
Left	TPJ	−54, −54, 22
Right	TPJ	54, −54, 22

**Table 3:** Main Effect of Peer Acceptance on Positive Emotions

Variable	Estimate	Beta	SE	t	*p*
(Intercept)	32.12	—	32.79	0.98	0.331
PeerAcceptance_bp	15.36	0.17	6.92	2.22	0.030
PeerAcceptance_wp	5.29	0.07	0.65	8.12	< 0.001
Age	0.96	0.03	2.11	0.46	0.650
Sex	5.30	0.11	3.82	1.39	0.170
Study Day	2.41	0.43	0.48	4.98	< 0.001
Weekend	0.19	0.54	0.81	2.39	0.021

*WP* = within-person variable (person-mean centered); *BP* = between-person variable (grand-mean centered or aggregated across time points). Significance was determined using a false discovery rate (FDR)-corrected threshold

**Table 4:** Results from Peer Acceptance X LTPJ to Positive Emotions Model

Variable	Estimate	Beta	SE	t	*p*
(Intercept)	34.10	−0.02	31.31	1.09	0.280
PeerAcceptance_bp	15.63	0.17	6.74	2.32	0.023
PeerAcceptance_wp	6.61	0.08	1.00	6.59	< 0.001
L TPJ	−0.01	−0.01	0.08	−0.20	0.846
Age	0.75	0.03	2.00	0.38	0.707
Sex	4.13	0.08	3.66	1.13	0.264
Study Day	0.20	0.06	0.08	2.41	0.019
Weekend	2.25	0.04	0.48	4.66	< 0.001
PeerAcceptance_wp: LTPJ	−0.14	−0.05	0.04	−3.23	0.002

*WP* = within-person variable (person-mean centered); *BP* = between-person variable (grand-mean centered or aggregated across time points). Significance was determined using a false discovery rate (FDR)-corrected threshold

**Table 5:** Results from Peer Acceptance X RTPJ to Positive Emotions Model

Variable	Estimate	Beta	SE	t	*p*
(Intercept)	33.17	−0.03	30.94	1.07	0.288
PeerAcceptance_bp	15.89	0.17	6.73	2.36	0.021
PeerAcceptance_wp	6.50	0.08	1.04	6.25	< 0.001
R TPJ	−0.04	−0.04	0.08	−0.51	0.612
Age	0.74	0.03	1.97	0.38	0.707
Sex	4.94	0.10	3.76	1.31	0.194
Study Day	0.20	0.07	0.08	2.43	0.019
Weekend	2.24	0.04	0.48	4.64	< 0.001
PeerAcceptance_wp: RTPJ	−0.13	−0.04	0.05	−2.61	0.011

*WP* = within-person variable (person-mean centered); *BP* = between-person variable (grand-mean centered or aggregated across time points). Significance was determined using a false discovery rate (FDR)-corrected threshold

**Table 6:** Results from Peer Acceptance X LACC to Positive Emotions Model

Variable	Estimate	Beta	SE	t	*p*
(Intercept)	35.34	−0.03	29.75	1.19	0.239
PeerAcceptance_bp	16.39	0.18	6.56	2.50	0.015
PeerAcceptance_wp	6.38	0.08	1.05	6.08	< 0.001
L ACC	−0.06	−0.07	0.06	−0.96	0.341
Age	0.93	0.03	1.92	0.49	0.629
Sex	4.34	0.09	3.52	1.23	0.223
Study Day	0.20	0.07	0.08	2.43	0.018
Weekend	2.26	0.04	0.48	4.67	0.239
PeerAcceptance_wp: LACC	−0.11	−0.04	0.04	−2.61	0.001

*WP* = within-person variable (person-mean centered); *BP* = between-person variable (grand-mean centered or aggregated across time points). Significance was determined using a false discovery rate (FDR)-corrected threshold

**Table 7:** Results from Peer Acceptance X RACC to Positive Emotions Model

Variable	Estimate	Beta	SE	t	*p*
(Intercept)	28.52	−0.03	30.14	0.95	0.348
PeerAcceptance_bp	15.70	0.17	6.55	2.40	0.019
PeerAcceptance_wp	6.29	0.08	1.07	5.86	< 0.001
R ACC	−0.08	−0.10	0.06	−1.31	0.194
Age	1.06	0.04	1.92	0.55	0.585
Sex	4.97	0.10	3.56	1.40	0.167
Study Day	0.20	0.07	0.08	2.44	0.018
Weekend	2.26	0.04	0.48	4.67	< 0.001
PeerAcceptance_wp: RACC	−0.08	−0.03	0.04	−2.06	0.043

*WP* = within-person variable (person-mean centered); *BP* = between-person variable (grand-mean centered or aggregated across time points). Significance was determined using a false discovery rate (FDR)-corrected threshold

**Table 8:** Main Effect of Peer Acceptance on Negative Emotions

Variable	Estimate	Beta	SE	t	*p*
(Intercept)	−1.38	—	26.39	−0.05	0.958
PeerAcceptance_bp	−7.83	−1.15	5.58	−1.40	0.165
PeerAcceptance_wp	−3.06	−0.56	0.42	−7.28	< 0.001
Age	1.75	0.08	1.70	1.03	0.307
Sex	−9.28	−0.25	3.07	−3.02	0.004
Study Day	−0.003	−0.00	0.071	−0.04	0.969
Weekend	−1.22	−0.03	0.31	−3.9	< 0.001

*WP* = within-person variable (person-mean centered); *BP* = between-person variable (grand-mean centered or aggregated across time points). Significance was determined using a false discovery rate (FDR)-corrected threshold

## Data Availability

De-identified raw data from this study are available on request from the corresponding author under a data sharing agreement. The data are not publicly available due to privacy and sensitive nature of the data (substance use in adolescents).
